# Interspecific Variations in Interplant Communication and Ecological Characteristics in Trees

**DOI:** 10.1002/ece3.70876

**Published:** 2025-01-17

**Authors:** Akira Yamawo, Tomika Hagiwara, Satomi Yoshida, Misuzu Ohno, Riku Nakajima, Yusuke Mori, Tamayo Hayashi, Hiroki Yamagishi, Kaori Shiojiri

**Affiliations:** ^1^ Department of Biology, Faculty of Agriculture and Life Science Hirosaki University Hirosaki Japan; ^2^ Center for Ecological Research Kyoto University Otsu, Shiga Japan; ^3^ Graduate School of Agriculture Kyoto University Kyoto Japan; ^4^ Faculty of Agriculture Ryukoku University Otsu Shiga Japan; ^5^ Faculty of Advanced Science and Technology Ryukoku University Otsu Shiga Japan; ^6^ Faculty of Agriculture and Life Science, The Shirakami Research Center for Environmental Sciences Hirosaki University Hirosaki Japan

**Keywords:** chemical communication, forest, forest ecology, herbivory, mutualism, mycorrhizal state, population density

## Abstract

Plants evolve diverse communication systems in adapting to complex and variable environments. Here, we examined the relationship between plant architecture, population density and inter‐plant communication within tree species. We tested the hypothesis that trees of species with complex architecture or high population density (high population density: HPD) communicate among conspecifics via volatiles. In addition, we hypothesize that states of mycorrhizal symbiosis (arbuscular mycorrhizal or ectomycorrhiza) which relation to population density can predict the development of interplant communication in trees. We tested induced defense as an indicator of communication in saplings of nine tree species with various complexities of architecture (number of leaves per shoot) and either low (low population density: LPD) or HPD, either exposed for 10 days to volatiles from a damaged conspecific or not exposed. We evaluated the number of insect‐damaged leaves and the area of leaf damage on these trees after 1 and 2 months in the field. Most exposed HPD trees had less leaf damage than controls. However, LPD trees did not differ in leaf damage between treatments. These results are partially supported by plant hormone analysis. In addition, the presence of inter‐plant communication was positively correlated with both the number of leaves per shoot (complexity of plant architecture) and population density. The analysis which combined results of previous studies suggests that states of mycorrhizal symbiosis predict the development of interplant communication; interplant communication is common in ectomycorrhiza species. These results suggest the importance of plant architecture and population density as well as state of mycorrhizal symbiosis in the development of interplant communications within tree species.

## Graphical abstract

How various factors have shaped the evolution of communication is a major concern of evolutionary ecology. We found that high population density related to mycorrhizal types and complexity of plant architecture positively correlated with interplant communication.

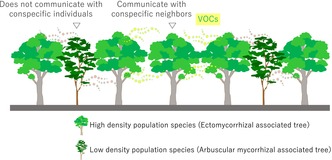

## 1 Introduction

Plants evolve diverse communication systems in adapting to complex and variable environments (Karban 2021). Interplant communication mediated by volatile organic compounds (VOCs) has been studied in many species (Heil and Karban 2010; Karban 2021; Karban, Yang, and Edwards 2014). Damaged plants release specific VOCs such as green leaf volatiles and terpenoids. VOCs from herbivore‐damaged plants activate the expression of resistance genes, prime resistance in surrounding undamaged plants (Heil and Karban 2010; Karban, Yang, and Edwards 2014; Yoneya and Takabayashi 2014; Takahashi, Shiojiri, and Yamawo 2021), and alter the behavior of herbivores, deterring herbivory of undamaged plants (Morrell and Kessler 2017; Arimura et al. 2000). Notably, VOCs from herbivore‐damaged plants convey important information for conspecifics, thus benefiting recipient plants. Although numerous studies focus on the mechanisms of plant–plant communication, it is unclear which ecological factors drive the development of plant–plant communication mediated by VOCs.

Within‐plant signaling may explain the origin of between‐plant communication (Heil and Bueno 2007; Heil and Karban 2010). VOCs play an important role as internal plant signals: studies of sagebrush, lima bean, poplar and blueberry show that VOCs released from damaged parts of plants induce or prime resistance in undamaged organs of the same individual (Heil and Karban 2010; Karban 2021). Within‐plant signaling by VOCs is faster than vascular signaling and is independent of plant anatomy (Farmer 2001; Orians 2005). These traits make VOCs capable of reaching leaves lacking direct vascular connections and leaves that are physically close but on different branches.

Population density is also an important factor in the development of plant–plant communication within species (Kobayashi and Yamamura 2007). In general, interplant communication is considered to benefit species that grow at a high density, because the effects of VOCs decrease with increasing distance, being effective in a few species within about 10 m of a defoliated tree (Tscharntke et al. 2001; Hagiwara et al. 2021). The population density correlate with mycorrhizal state. Plant–soil feedback (PSF), mediated through interaction with microbial communities, regulates tree population density (e.g., Bennett et al. 2017; Delavaux et al. 2023). The direction (positive or negative for conspecific individuals) of PSF effects depends on the state of mycorrhizal symbiosis. Bennett et al. (2017) showed that arbuscular mycorrhizal (AM)‐associated trees had negative effects on growth or survival of conspecific seedlings (negative soil–plant feedback), whereas ectomycorrhizal (ECM)‐associated trees had positive effects (positive soil–plant feedback). These PSF effects correlated with population density in the field. Thus, mycorrhizal type may predict the development of plant–plant communication through population density.

We hypothesized that complex plant architecture (high number of leaves per shoot), high population density (HPD), mycorrhizal state (AM or EcM) or all correlate with development of communication between conspecific individuals. We conducted an experiment in two study fields using 9 tree species with various architectures and population densities in natural conditions (Table 1). Thirty saplings of each species were exposed for 10 days to conspecific neighbors that had been damaged by clipping. As controls, another 30 saplings were not exposed. All saplings were then set in natural fields, and we evaluated the number of insect‐damaged leaves at 1 month and the area of leaf damage at 2 months. We also compared two plant defense hormones, jasmonic acid (JA) and salicylic acid (SA), between treatments at 10 days. Finally, we analyzed relationships between complexity of plant architecture, population density or mycorrhizal state and interspecific variations in outcomes of interplant communication.

**TABLE 1:** Population densities in natural condition in each study species.

Study species	Mychorrhizal type	*n*	Population density (/10,000 m^2^)
*Aesculus turbinata*	AM	5	30.2 ± 19.1
*Magnolia obovate*	AM	19	23.0 ± 32.9
*Prunus jamasakura*	AM	10	30.4 ± 18.7
*Viburnum furcatum*	AM	6	28.2 ± 31.1
*Viburnum wrightii*	AM	1	2
*Betula platyphylla*	EcM	6	84.9 ± 124.1
*Pinus densiflora*	EcM	2	36.0 ± 31.1
*Quercus crispula*	EcM	3	80.0 ± 131.63
*Quercus serrata*	EcM	15	162.9 ± 358.0

*Note:* Data are based on the “Monitoring Sites 1000” program of nationwide long‐term monitoring of tree communities in Japan (Ishihara et al. 2010).

Abbreviations: AM, arbuscular mycorrhiza; ECM, ectomycorrhizal mycorrhiza; *n*, number of sites.

## 2 Materials and Methods

### 2.1 Experimental Species

We selected nine plant species growing dominantly around an experimental field in Shirakami Natural Science Park, Hirosaki University (40°52′ N, 140°22′ E), and in Ryukoku University Forest (34°96′ N, 135°94′ E): five low population density (LPD) species (*Magnolia obovata*, *Cerasus jamasakura*, *Viburnum dilatatum* , *Viburnum furcatum*, and *Aesculus turbinata* ) which are AM‐associated species and four HPD species ( *Pinus densiflora* , *Betula platyphylla* , *Quercus crispula*, and *Quercus serrata* ) which are EcM‐associated species (Table 1; Ishihara et al. 2010). Population density of *Pinus densiflora* was middle value, but we include the HPD species because this species was more dominant decades before the pest epidemic. Although this study did not directly analyze mycorrhizae, the type of mycorrhizal symbiosis is fixed at the genus level (Tedersoo, Bahram, and Zobel 2020). Therefore, we relied on information identified in previous studies (Soudzilovskaia et al. 2020).

Because plant defenses for future growth are often developed at the seedling stage (Barton and Koricheva 2010), we purchased sixty 3‐ to 5‐year‐old saplings of each species from a local nursery in Fukuoka City in November 2020. None had large lateral branches and all were about 50 cm tall. *Betula platyphylla* is known to communicate with conspecific neighbors through VOCs (Rieksta et al. 2020). The plants were grown in a common natural soil.

### 2.2 Plant Culture

In April and May 2021, all saplings were individually planted in plastic pots (15 × 15 × 25 cm) containing 50% tuff loam and 50% humus. They were maintained in a greenhouse at each university which maintains natural temperature. The experiment began when new leaves reached the three‐ or four‐leaf stage, in early spring (April).

### 2.3 Plant Architecture

VOCs released from damaged parts of plants induce or prime resistance in undamaged organs, often affecting other leaves of the same individual (Heil and Karban 2010; Karban 2021). Therefore, it is believed that the development of VOCs signaling is influenced by the complexity of the plant architecture. In this study, we assessed the complexity of plant architecture by counting all leaves per 50 cm of each plant prior to the experiment. There are various indicators of the complexity of plant architecture, but in the context of interplant communication where transmission of information from damaged leaves to undamaged ones is crucial, leaf count simply signifies branching points for information transmission to destination. Hence, it is considered suitable as an indicator of architecture complexity.

### 2.4 Experimental Design

Saplings of each species were randomly assigned to either the control group (no exposure to VOCs, *n* = 30) or the exposure treatment group (exposed to VOCs, *n* = 30). The two groups were held in different areas more than 10 m away and separated by a glass wall at each site. The floor was concrete and there were no other plants around. In the exposure area, we placed five conspecific “emitter” plants on which half of every leaf was clipped, located 60 cm apart. Around each emitter plant we arranged six experimental plants 30 cm away. Some plants release specific VOCs against specific herbivores (Karban 2021; Mann et al. 2021). However, as we used artificial clipping by scissors, our experiment can reveal interspecific variations in plant–plant communication in response to common leaf damage such as by generalist herbivores. In the control area, the experimental plants were placed around five undamaged plants. After 10 days, all plants were set in natural conditions for each species (Shirakami Natural Science Park: *M. obovata* , *V. dilatatum* , *A. turbinata* , *B. platyphylla* , *Q. crispula*; Ryukoku University Forest: *Cerasus jamasakura*, *Viburnum wrightii*, *Pinus densiflora* , *Quercus serrata* ) for 2 months. The species composition at each site was determined from the natural vegetation there. The sites were open, surrounded by deciduous broad‐leaved trees and pine trees. Pots were placed randomly 60 cm apart. The plants were watered every 2 days.

In recent years, it has been suggested that vibrations and sounds, in addition to VOCs, also contribute to interplant communication (Son et al. 2024). However, since many of these effects have been demonstrated under experimental conditions with minimal noise, the induced defense responses observed in neighboring plants in this study were determined to be the result of interplant communication mediated by VOCs.

### 2.5 Evaluation of Defense Induction

As indicators of defense induction, we counted the number of insect‐damaged leaves at 1 month and measured the area of leaf damage at 2 months after treatments. We also compared two plant defense hormones, JA and SA, between treatments at 10 days after treatment.

### 2.6 Number of Damaged Leaves and Area of Leaf Damage

The timing of induced defense expression may vary between species; therefore, in this experiment, we investigated herbivory at two time points: 1 month and 2 months after the initial treatment. In all species except *P. densiflora* , at 1 month, on 10 or 12 September, 2021, we measured herbivory by counting the leaves with any visible damage caused by insect herbivores on all leaves in both treatments in field. We have used this presence/absence measure of herbivory in our many previous works and found that it correlates with the percentage of leaf area removed. In *Pinus densiflora* , we counted the leaves damaged that had brown marks as leaf damage by stink bugs, because they had no leaf‐area loss and had only dark stab marks.

At 2 months, all leaves were collected and scanned on an image scanner (PM‐850; Seiko Epson Corp., Suwa, Japan). The area of each leaf was subsequently measured in Scion Image photo‐image analysis software (Scion Image; Scion Corp., Frederick, MD, USA). The area that had been consumed by herbivores was estimated by comparison with an uninjured leaf of equal size. *Cerasus jamasakura* was excluded from this analysis because of a technical mistake when the leaves were collected. In *Pinus densiflora* , we counted the leaves damaged by stink bugs per randomly selected 500 leaves, because they had no leaf‐area loss and had only dark stab marks.

### 2.7 Plant Hormones

To determine any response induced in leaves exposed to VOCs, we analyzed the contents of JA and SA in leaves by liquid chromatography–tandem mass spectrometry (LC–MS) according to Ozawa et al. (2017) in 1–2 mm of leaf cut from each plant with scissors after 10 days' exposure in both treatments.

Leaves (ca. 0.5 g) were immediately frozen in liquid nitrogen, homogenized in ethyl acetate (2.5 mL), and spiked with 10 ng of d2‐JA (Tokyo Chemical Industries Co., Tokyo, Japan) and 1 ng of d4‐SA (C/D/N Isotopes, Pointe‐Claire, QC, Canada) as internal standards. After centrifugation of the mixture at 2300×*g* for 10 min at 4°C, 1 mL of supernatant was transferred to a 1.5‐mL tube and then evaporated to dryness under vacuum. The residue was suspended in 50 μL of 70% methanol/water (*v*/*v*) and centrifuged, and the supernatant was analyzed by LC–MS/MS (LCMS‐8050, Shimadzu, Kyoto, Japan). Analytes were separated by high‐performance liquid chromatography through a Mightysil RP‐18 GP column (100 × 2.0 mm, 3 μm particle size, Kanto Chemical, Tokyo, Japan) at a flow rate of 200 μL min^−1^ with a linear gradient (0.1% formic acid aq. “A” and methanol “B”; 5%–95% B/(A + B) for 16 min). Concentrations of JA, d2‐JA, SA, and d4‐SA were determined by multiple reaction monitoring (MRM). The monitored mass transitions were *m/z* 209 to *m/z* 59 for JA, *m/z* 211 to *m/z* 59 for d2‐JA, *m/z* 137 to *m/z* 93 for SA, and *m/z* 141 to *m/z* 97 for d4‐SA. The conditions for MS were optimized for MRM with authentic d2‐JA and JA (Tokyo Chemical Industries), d4‐SA (C/D/N Isotopes), and SA (Wako Pure Chemical Industries, Osaka, Japan). Samples with technical mistakes were removed from all data analysis.

### 2.8 Data Analysis

All data were analyzed in R v. 4.1.0 software (R Development Core Team 2021). All data tested the statistical assumptions of normality and homoscedasticity according to the Kolmogorov–Smirnov test and *F*‐test, and analyses performed depended on the dataset structure. All tests were two tailed, with *p* < 0.05 considered significant.

### 2.9 Number of Damaged Leaves, Area of Leaf Damage, and Plant Hormones

The number of damaged leaves and area of leaf damage were analyzed by general linear models (GLMs) with Poisson or gamma distributions, and tested by chi‐squared test. Damage of *P. densiflora* by stink bugs was analyzed by a GLM with a Poisson distribution, and tested by chi‐squared test. Number of damaged leaves and area of leaf damage or damage by stink bugs were included as response variables, and exposure treatment, mycorrhizal types and their interaction were included as explanatory variables. The total number of leaves was included as a covariate. When there was a difference in exposure treatment between mycorrhizal types, we analyzed the effect of exposure treatment on leaf damage by mycorrhizal type separately.

Concentrations of plant hormones were compared between control and VOC‐exposed plants by a GLM with a negative‐binomial distribution, and tested by chi‐squared test. A false discovery rate correction for multiple comparisons was then applied.

The relationships between presence of plant–plant communication and population densities were analyzed by GLMs with a binomial distribution, and tested by chi‐squared test. The presence or absence of plant–plant communication was included as a response variable, and population density was included as an explanatory variable.

### 2.10 Relationships Between Plant Architecture and Interplant Communication

We focused on the 8 angiosperm trees, because gymnosperms, including *Pinus densiflora* , develop a significantly different plant architecture from angiosperm species, such as their multitudinous needle leaves. The number of leaves in each species and the presence of plant–plant communication was analyzed by GLM with a binomial distribution with a log‐link function, and was tested by chi‐squared test.

### 2.11 Phylogenetic Correlation Between Mycorrhizal State and Interplant Communication

We searched studies in Google Scholar (https://scholar.google.com/) for papers in English and Japanese with the terms “plant–plant communication” and “tree”. We also used data on presence or absence of plant–plant communication that were collected from the list by Heil and Karban (2010). Our database contains information on the mycorrhizal types, and plant–plant communication of 21 species in 12 genera in 8 families. To prepare a phylogenetic tree, species names were standardized against Plant List 1.1 (http://www.theplantlist.org/), and membership in higher taxonomic groups was standardized against APG IV (Angiosperm Phylogeny Group 2016).

The phylogenetic tree was constructed in PhytoPhylo tree software (Qian and Jin 2016), being based on the phylogeny generated by Zanne et al. (2014) and updated by Qian and Jin ( 2016). The final phylogenetic tree had 21 tip labels and 19 internal nodes in scenario 3, the accuracy of which has been verified (Figure S1).

The PhytoPhylo phylogenetic tree was used for phylogenetic generalized least squares (pGLS). We used Pagel's λ correlation structure to incorporate the phylogenetic information into the models. The models were constructed by using the corPagel function in the “ape” package (Paradis 2019) with the amount of phylogenetic signal in the data estimated by the restricted maximum likelihood approach. The presence or absence of plant–plant communication was included as a response variable, and mycorrhizal type was included as an explanatory variable. A dataset from the Global Biodiversity Information Facility (https://www.gbif.org) was used to compile information on tree mycorrhizal type (AM or ECM) (Soudzilovskaia et al. 2020).

## 3 Results

### 3.1 Effects of VOCs Exposed Treatment on Leaf Damage

**FIGURE 1:**
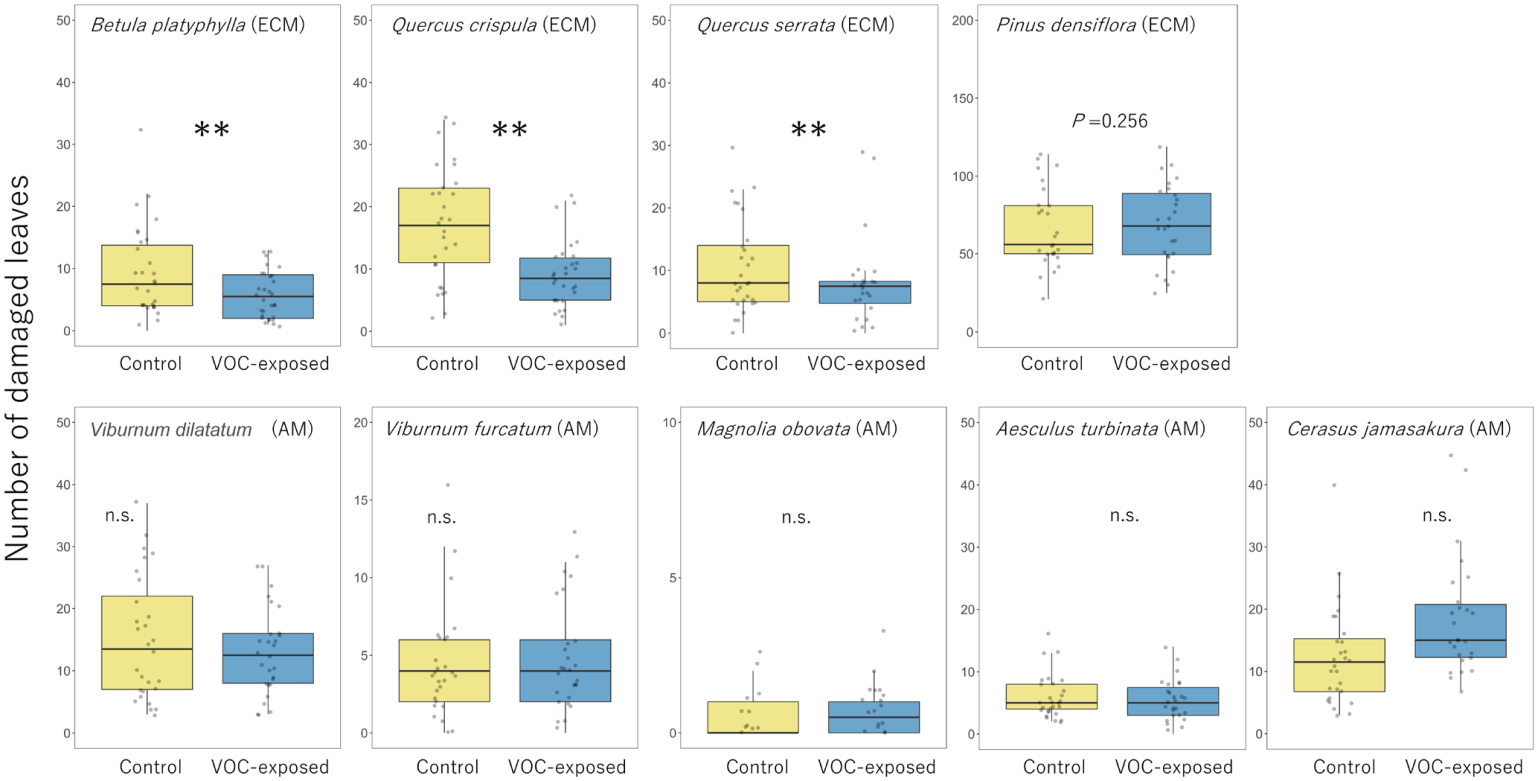
Number of damaged leaves at 1 month in control and exposed plants. Values in *Pinus densiflora* are the number of damage marks caused by stink bugs. **p < 0.05.

**FIGURE 2:**
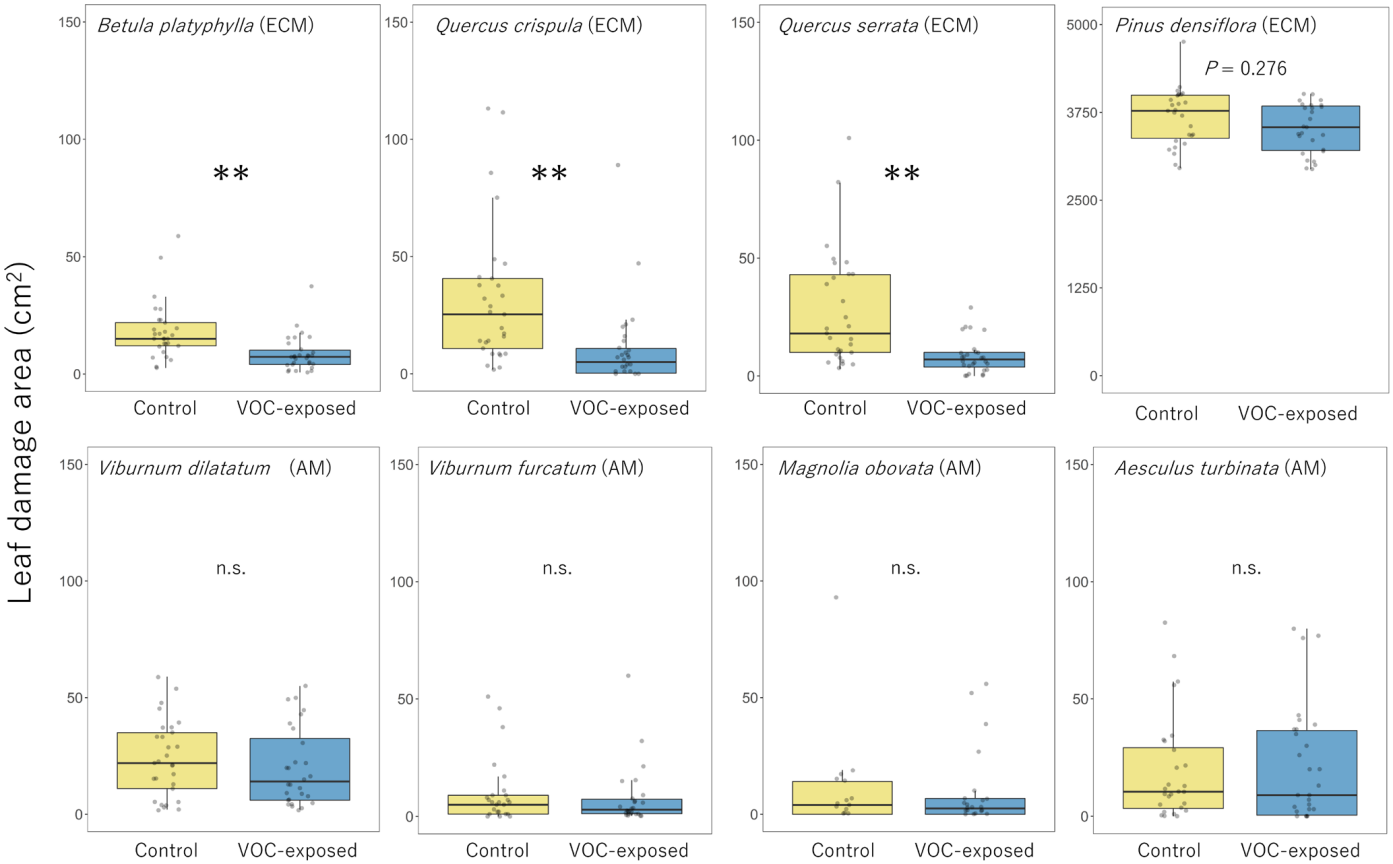
Area of leaf damage at 2 months in control and exposed plants. Values in *Pinus densiflora* are the number of damage marks caused by stink bugs. **p < 0.05.

Effects of exposure treatment on leaf damage differed clearly among population density at both 1 month (*χ* ^2^ = 81.10, *p* < 0.001; Figure 1) and 2 months (*χ* ^2^ = 14.89, *p* < 0.001; Figure 2). Both number of damaged leaves and area of leaf damage in exposed HPD species— *Betula platyphylla* , *Quercus crispula* and *Q. serrata* (but not *Pinus densiflora* )—were significantly smaller than those in control trees (1 month, *χ* ^2^ = 93.06, *p* < 0.001; 2 months, *χ* ^2^ = 52.01, *p* < 0.001). However, they did not differ between treatments in LPD species, *Magnolia obovata*, *Cerasus jamasakura*, *Viburnum dilatatum* , *Viburnum wrightii*, and *Aesculus turbinata* or *Pinus densiflora* (LPD:1 month, *χ* ^2^ = 0.86, *p* = 0.35; 2 months, *χ* ^2^ = 0.34, *p* = 0.56; *P. densiflora* : 1 month, *χ* ^2^ = 0.23, *p* = 0.63; 2 months, *χ* ^2^ = 1.30, *p* = 0.25).

The plant hormone assays also showed evidence of interplant communication in two species. Exposure increased JA in *B. platyphylla* (Table S1), supporting the results of reduced leaf damage. On the other hand, we could not find differences in plant hormones in *Q. crispula* or *Q. serrata* , despite these species' significant differences in leaf damage between treatments. This discrepancy suggests that the defense response was induced after the hormone measurements (10 days from start of exposure treatment) in *Quercus*. In addition, JA and SA contents in *C. jamasakura* were significantly higher in exposed trees, despite no difference in leaf damage (Table S1). This suggests that induced responses in *C. jamasakura* are too small to affect leaf damage by insect pests. JA and SA contents did not differ between treatments in other species. These results mean that the importance of carefully assessing plant–plant communication in a variety of ways and differences in induction timing.

The presence/absence of interplant communication within our nine tree species was positively correlated with population density (*C. jamasakura* include as non‐communicated: *χ* ^2^ = 11.5, *p* < 0.001; *C. jamasakura* include as communicated: *χ* ^2^ = 7.3, *p* < 0.001).

### 3.2 Plant Architecture and Interplant Communication

HPD species had significantly more leaves than LPD species (HPD, 88.4 ± 31.6; LPD, 45.9 ± 26.1, *χ* ^2^ = 46.4, *p* < 0.001). Numbers of leaves were positively correlated with the presence of plant communication (*C. jamasakura* included as non‐communicated: *χ* ^2^ = 6.1, *p* = 0.014; *C. jamasakura* included as communicated: *χ* ^2^ = 4.3, *p* = 0.036).

### 3.3 Phylogenetic Correlation Between Mycorrhizal State and Interplant Communication

We found 12 previous studies of interplant communication in different tree species (Data S3). All tree species belonged to plant families which could associate with ECM‐mycorrhizal symbiosis, except for *Acer saccharum* . Combining our results with previously published data, we found marginal or significant phylogenetic correlations of type of mycorrhizal symbiosis with interplant communication (*C. jamasakura* include as non‐communicated: λ = 1, *F* = 15.76, *p* < 0.001; *C. jamasakura* include as communicated: λ = 1, *F* = 3.53, *p* = 0.075).

## 4 Discussion

Overall, interplant communication between conspecific individuals was more common in species with a more complex plant architecture, supporting the hypothesis that it originated from within‐plant signaling (Heil and Bueno 2007; Heil and Karban 2010). Population densities and mycorrhizal state were also positively correlated with the development of interplant communication, supporting our prediction. These findings suggest an evolutionary process driving plant–plant communication in HPD tree species, involving: (1) the development of complex architectures, (2) the establishment of within‐plant signaling through VOCs, and (3) the emergence of interplant communication among conspecifics in HPD tree species. However, our study was limited to just 9 species. Therefore, to more comprehensively understand the evolution of interplant communication, future investigations should encompass a wider array of tree species, enabling an exploration of the relationships between plant architecture complexity or within‐plant signaling and population density.

There are several other limitations to understanding the development of interplant communication from our experiment. First, we have not fully tested the communication hypothesis, because plants signal not only leaf damage but also abiotic stress (Landi 2020; Karban 2021), which LPD species may also recognize. Importantly, note that our experiment focused only on plant–plant communication based on artificial leaf damage. Second, our study species have a large phylogenetic bias because we chose them from among vegetation on site; for example, two of the four HPD trees were *Quercus* species. Therefore, future studies should focus on communication not only of leaf damage but also of abiotic stress among more phylogenetically diverse species.

Despite these limitations, our results suggest that the interspecific variation of interplant communication is associated with complexity of plant architecture and population density. Given the susceptibility of population density to various ecological factors such as microtopography and disturbance, irrespective of plant architecture complexity, it may be challenging to thoroughly assess the true significance of the role it has played in the evolution of plant communication on broader scales. States of mycorrhizal symbiosis can influence the population density of tree species through plant–soil feedback (PSF) effects (Bennett et al. 2017; Delavaux et al. 2023). The direction (positive or negative) of PSF effects between conspecific individuals depends on the type of mycorrhizal symbiosis: arbuscular mycorrhizal (AM)‐associated trees have negative effects on growth or survival of conspecific seedlings (negative plant–soil feedback), whereas ectomycorrhizal (ECM)‐associated trees have positive effects (positive PSF), which correlate with population density in the field (Bennett et al. 2017). In addition, ECM types are more likely to evolve short‐distance seed dispersal (Yamawo and Ohno 2024), which should promote HPD and thus the evolution of interplant communication. In fact, all species that showed interplant communication in our experiments were ECM species (Table 1). Moreover, our analysis suggests that interplant communication is common in ECM species (Figure S1): specifically, all species except for *Acer saccharum* belong to families that can associate with ECM‐mycorrhizal symbiosis. Considering our results in combination with previously published data suggests associations of state of mycorrhizal symbiosis with interplant communication. Thus, mycorrhizal state may predict the development of interplant communication within species (Figure S2). It may be beneficial to test the effects of population density or mycorrhizal state in future studies of the evolution of interplant communication in trees.

## Author Contributions

**Akira Yamawo:** conceptualization (equal), data curation (equal), formal analysis (equal), funding acquisition (equal), investigation (equal), methodology (equal), writing – original draft (equal), writing – review and editing (equal). **Tomika Hagiwara:** data curation (equal), formal analysis (equal), investigation (equal), resources (equal), visualization (equal), writing – review and editing (equal). **Satomi Yoshida:** investigation (equal). **Misuzu Ohno:** data curation (equal), formal analysis (equal). **Riku Nakajima:** investigation (equal). **Yusuke Mori:** investigation (equal), writing – review and editing (equal). **Tamayo Hayashi:** data curation (equal), investigation (equal), resources (equal). **Hiroki Yamagishi:** investigation (equal). **Kaori Shiojiri:** conceptualization (equal), data curation (equal), formal analysis (equal), funding acquisition (equal), investigation (equal).

## Conflicts of Interest

The authors declare no conflicts of interest.

## Supporting information


**Data S1.** One month.

**Data S2.** Two month.

**Data S3.** Supplementary data.

**Figure S1.** Time‐calibrated phylogeny of the 21 plant species used in this study and previous studies (supplementary data) provided by Scenario 3 of S.PhyloMaker. Branch color denotes mycorrhizal type (red, AM; blue, ECM). Colors of inner and outer labels ringing the tree indicate communication ability (red, absence; blue, presence). *Cerasus jamasakura* is colored in both red and blue, because this species had different results between herbivory and plant hormones‐based estimation. A dataset from the Global Biodiversity Information Facility (https://www.gbif.org) was used to compile information on tree mycorrhizal type (AM or ECM) (Soudzilovskaia et al. 2020). The scale bar represents 40 million years. Phylogenetical analysis show the marginal or significant phylogenetic correlations of type of mycorrhizal symbiosis with plant–plant communication (*C. jamasakura* include as non‐communicated: λ = 1, *F* = 15.76, *p* < 0.001; *C. jamasakura* include as communicated: λ = 1, *F* = 3.53, *p* = 0.075).
**Figure S2.** Conceptual diagram of our hypothesis. ECM symbiosis, which is positively associated with tree population density, promotes communication between conspecific individuals.

Table S1.

## Data Availability

All datasets are available from the Supporting Information files. *Code availability statement*: The computer code is available from the corresponding author on reasonable request.
