# Supplementary material for: Interspecific Variations in Interplant Communication and Ecological Characteristics in Trees
Source: Ecol Evol. 2025 Jan 17;15(1):e70876. doi: 10.1002/ece3.70876 (PMC11739611; doi:10.1002/ece3.70876)
Supplement: Supplementary file 4 — Figure S1. Time‐calibrated phylogeny of the 21 plant species used in this study and previous studies (supplementary data) provided by Scenario 3 of S.PhyloMaker. Branch color denotes mycorrhizal type (red, AM; blue, ECM). Colors of inner and outer labels ringing the tree indicate communication ability (red, absence; blue, presence). Cerasus jamasakura is colored in both red and blue, because this species had different results between herbivory and plant hormones‐based estimation. A dataset from the Global Biodiversity Information Facility (https://www.gbif.org) was used to compile information on tree mycorrhizal type (AM or ECM) (Soudzilovskaia et al. 2020). The scale bar represents 40 million years. Phylogenetical analysis show the marginal or significant phylogenetic correlations of type of mycorrhizal symbiosis with plant–plant communication (C. jamasakura include as non‐communicated: λ = 1, F = 15.76, p < 0.001; C. jamasakura include as communicated: λ = 1, F = 3.53, p = 0.075). Figure S2. Conceptual diagram of our hypothesis. ECM symbiosis, which is positively associated with tree population density, promotes communication between conspecific individuals. [file ECE3-15-e70876-s004.pptx]

## Slide 1
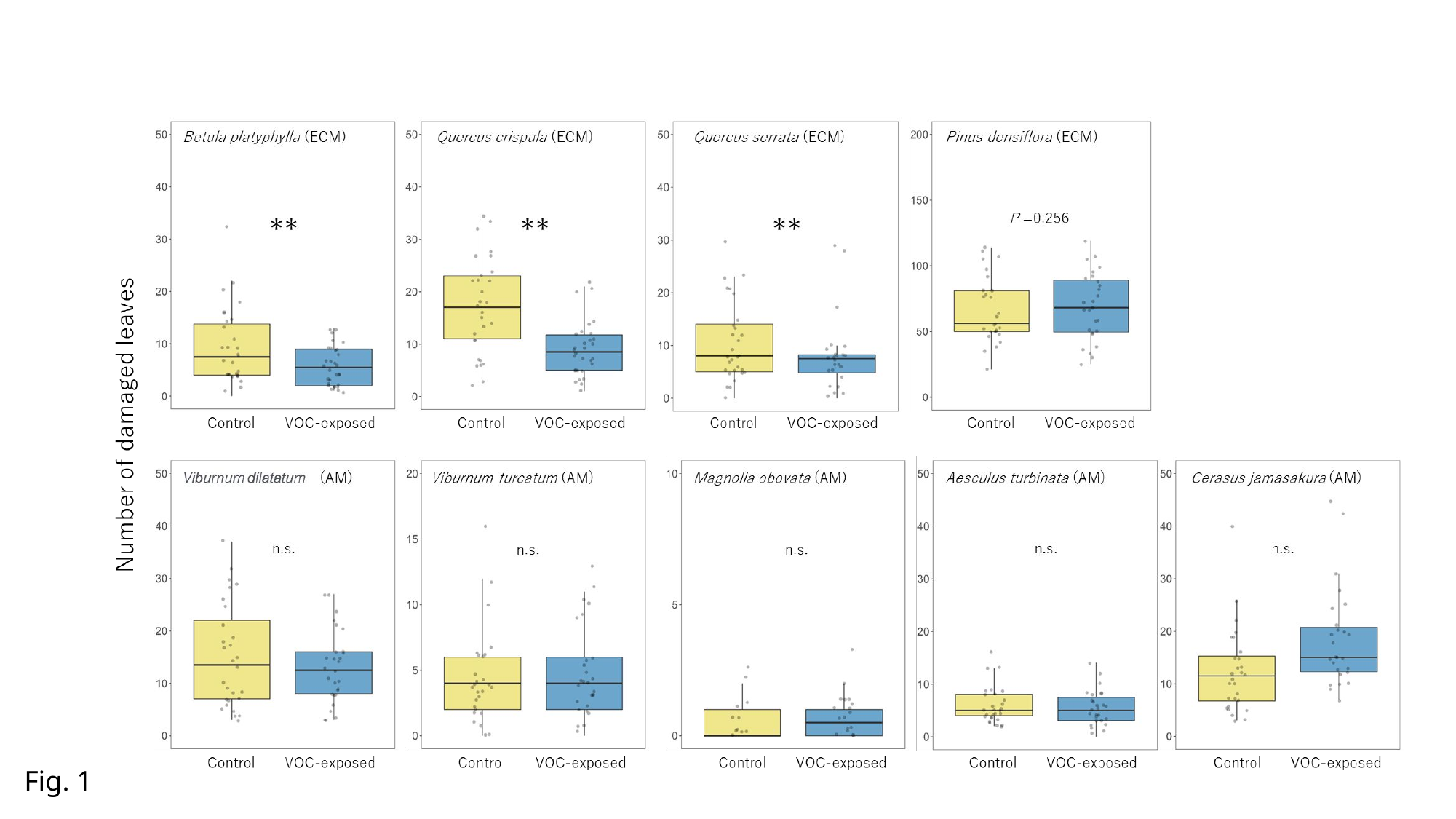

Fig. 1

## Slide 2
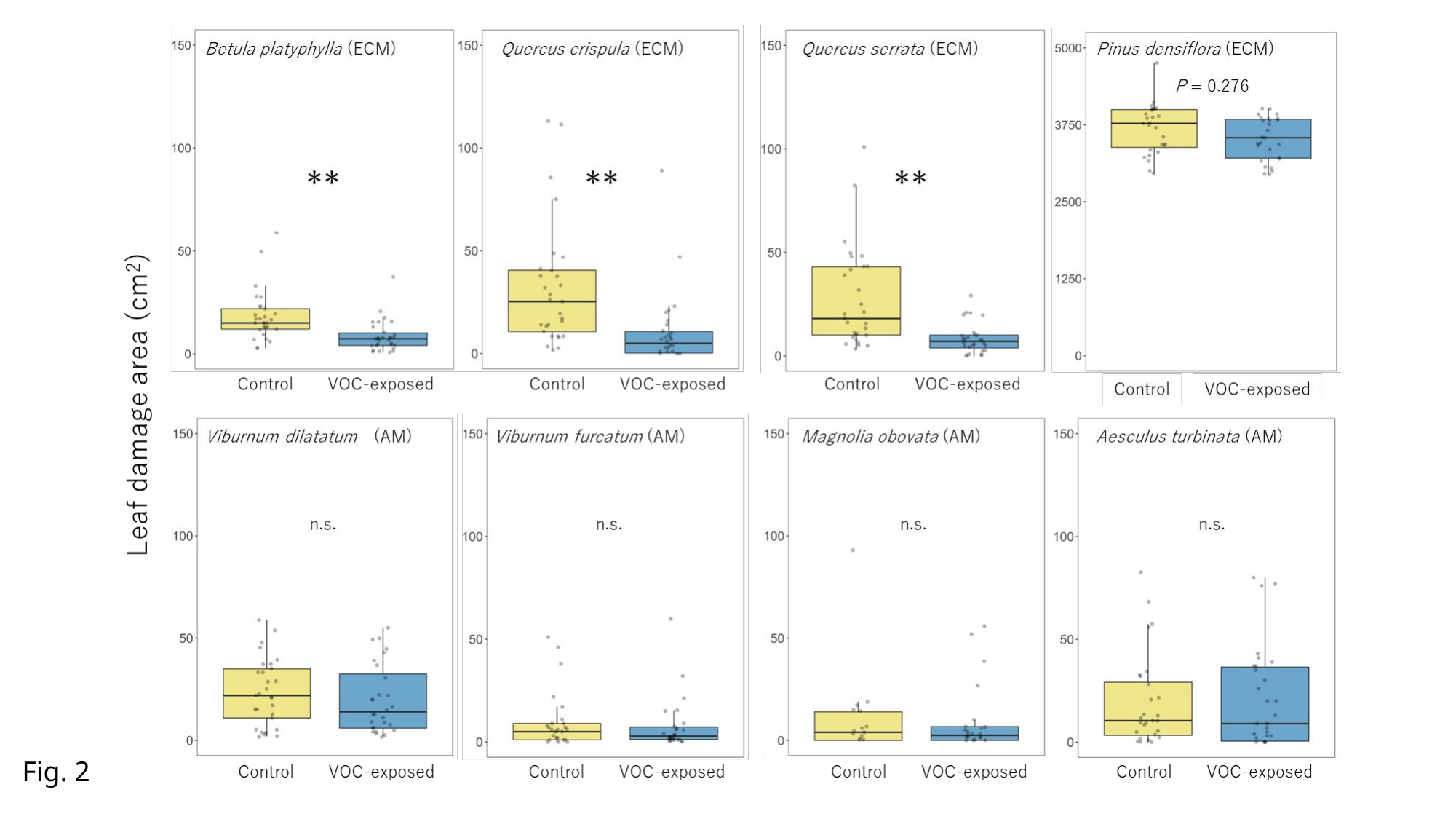

Fig. 2

## Slide 3
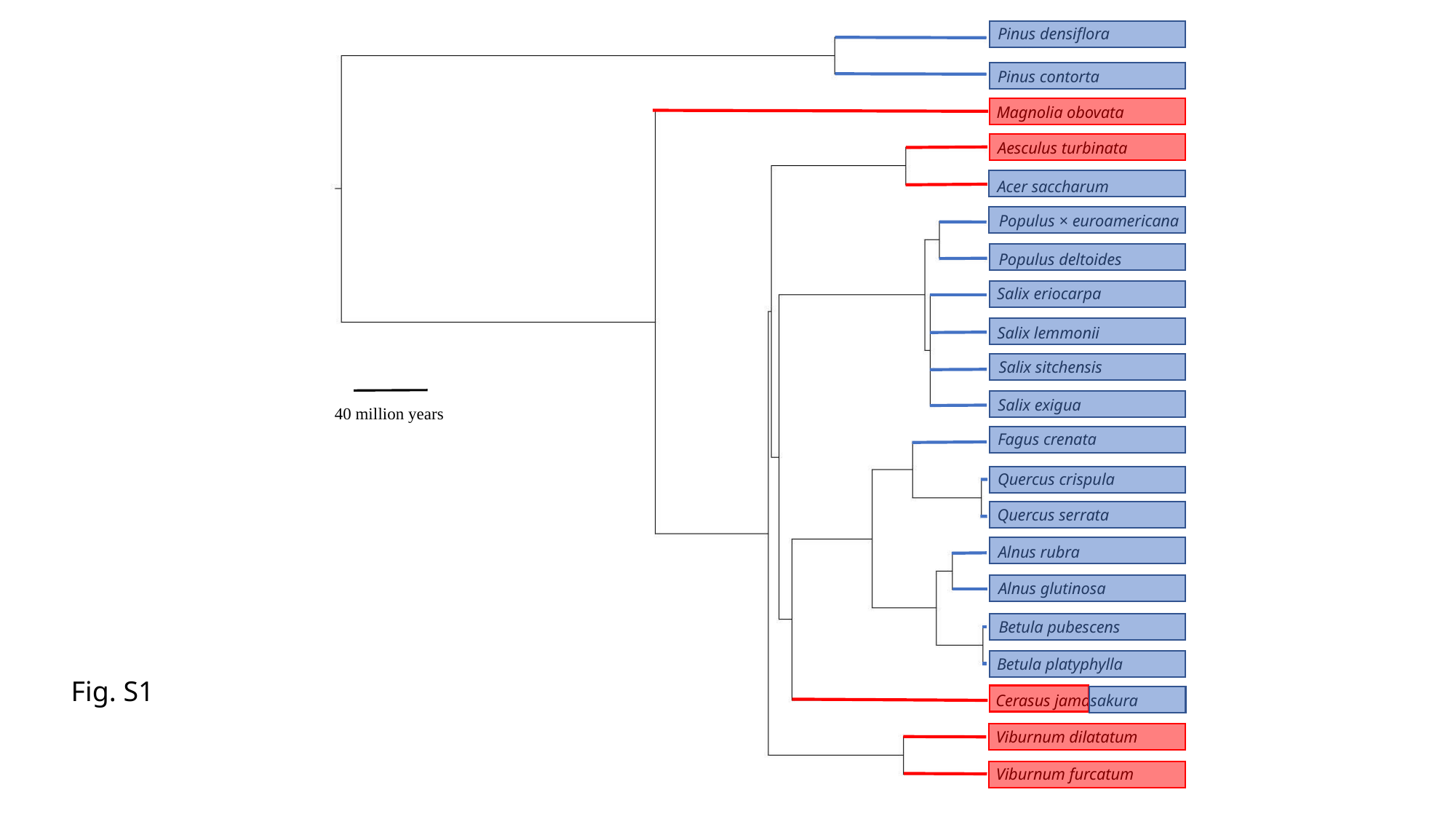

Pinus densiflora
Pinus contorta
Magnolia obovata
Aesculus turbinata
Acer saccharum
Populus × euroamericana
Populus deltoides
Salix eriocarpa
Salix lemmonii
Salix sitchensis
Salix exigua
Fagus crenata
Quercus crispula
Quercus serrata
Alnus rubra
Alnus glutinosa
Betula pubescens
Betula platyphylla
Cerasus jamasakura
Viburnum dilatatum
Viburnum furcatum
40 million years
Fig. S1

## Slide 4
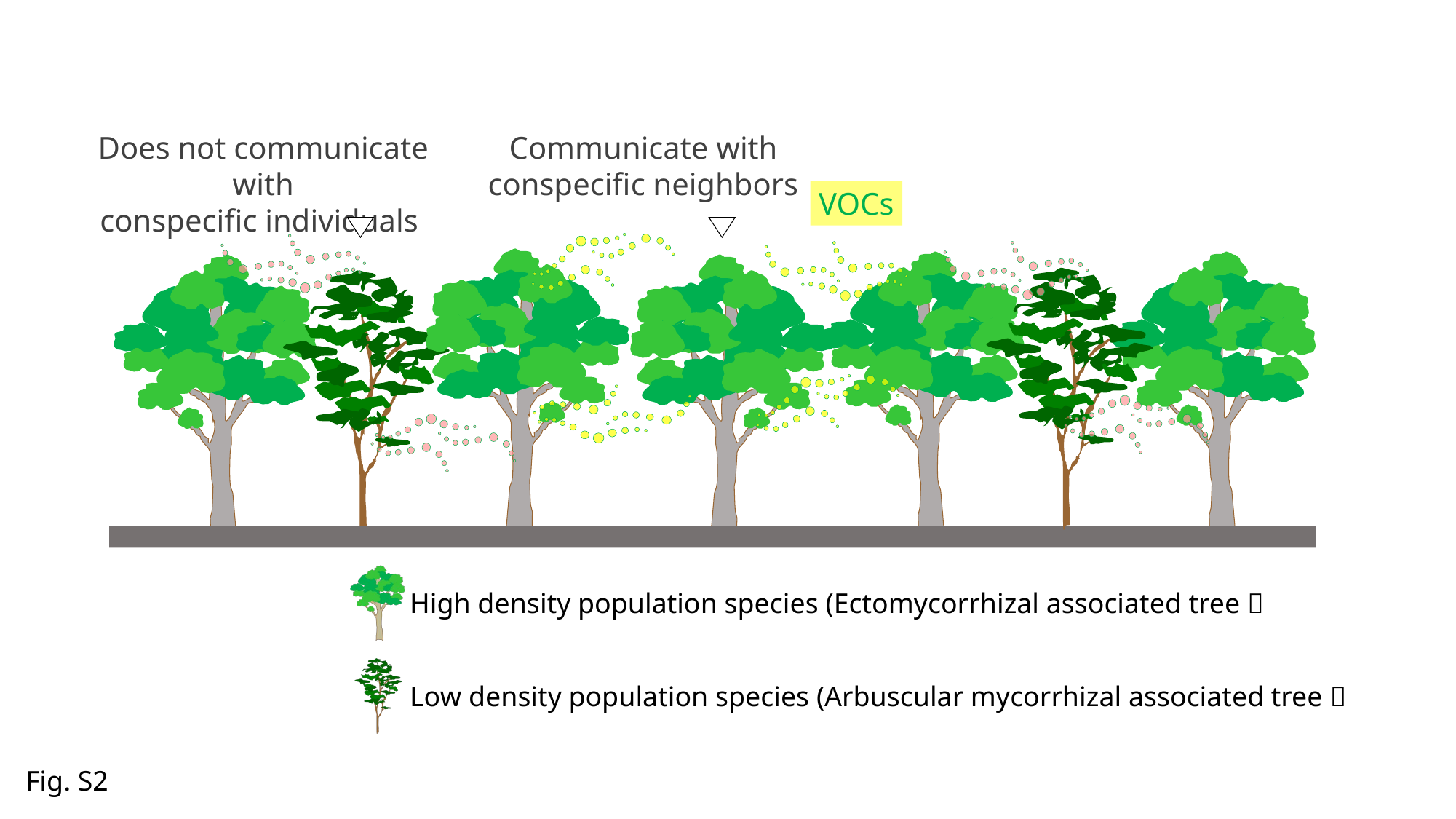

Does not communicate with
conspecific individuals
Communicate with conspecific neighbors
VOCs
High density population species (Ectomycorrhizal associated tree）
Low density population species (Arbuscular mycorrhizal associated tree）
Fig. S2
