# Supplementary material for: Interspecific Variations in Interplant Communication and Ecological Characteristics in Trees
Source: Ecol Evol. 2025 Jan 17;15(1):e70876. doi: 10.1002/ece3.70876 (PMC11739611; doi:10.1002/ece3.70876)
Supplement: Supplementary file 5 — Table S1. [file ECE3-15-e70876-s005.docx]

**Table S1** Contents of plant defence hormones in control and exposed plants.

|  | Jasmonic acid | | | |  | Salicylic acids | | | |
| --- | --- | --- | --- | --- | --- | --- | --- | --- | --- |
| Plant species | Control | Communicated | *nb* | *P-value* |  | Control | Communicated | *nb* | *P-value* |
| *Quercus serrata* | 43.59 ±40.50 | 33.86 ±21.20 | 58.96 | 0.24 |  | 67.99 ±62.66 | 87.85 ±62.14 | 60.43 | 0,52 |
| *Pinus densiflora* | - | - | - | - |  | 423.67 ±274.83 | 464.86 ±62.14 | 55.08 | 0.83 |
| *Quercus crispula* | 4.77±5.39 | 4.70 ±4.49 | 63.8 | 0.95 |  | 27.52 ±27.21 | 18.72 ±28.09 | 65.86 | 0.41 |
| *Betula platyphylla* | 26.56±26.71 | 41.46 ±28.97 | 1181.9 | **0.0008** |  | 27.91 ±5.24 | 26.80±17.25 | 60.47 | 0.83 |
|  |  |  |  |  |  |  |  |  |  |
| *Magnolia obovata* | 8.73 ±30.32 | 18.91 ±20.53 | 37.61 | 0.18 |  | 13.56 ±10.92 | 11.08 ±12.96 | 35.88 | 0.83 |
| *Aesculus turbinata* | 21.70 ±18.98 | 27.83 ±26.87 | 61.75 | **0.24** |  | 2.44 ±3.53 | 3.18 ±3.24 | 55.32 | **0.52** |
| *Cerasus jamasakura* | 2.31 ±1.36 | 10.94±7.96 | 13.48 | 0.004 |  | 2.20 ±1.16 | 21.06 ±3.25 | 15.69 | 0.009 |
| *Viburnum dilatatum* | 69.96 ±53.28 | 45.96 ±21.89 | 35.02 | 0.16 |  | 41.48±13.76 | 38.24 ±5.16 | 37.66 | 0.83 |
| *Viburnum furcatum* | 1.42 ±1.34 | 0.80 ±0.69 | 27.21 | 0.22 |  | 3.29±1.17 | 3.06 ±1.46 | 13.67 | 0.83 |

**Bold** indicates *P* < 0.05.
